# Pathogenetic, Prognostic, and Therapeutic Role of Fatty Acid Synthase in Human Hepatocellular Carcinoma

**DOI:** 10.3389/fonc.2019.01412

**Published:** 2019-12-11

**Authors:** Li Che, Panagiotis Paliogiannis, Antonio Cigliano, Maria G. Pilo, Xin Chen, Diego F. Calvisi

**Affiliations:** ^1^Department of Bioengineering and Therapeutic Sciences and Liver Center, University of California, San Francisco, San Francisco, CA, United States; ^2^Department of Medical, Surgical and Experimental Medicine, University of Sassari, Sassari, Italy; ^3^Institut für Pathologie, Universität Regensburg, Regensburg, Germany

**Keywords:** hepatocellular carcinoma, *de novo* lipogenesis, FASN, tumor metabolism, precision medicine

## Abstract

Hepatocellular carcinoma (HCC) is one of the most common solid tumors worldwide, characterized by clinical aggressiveness, resistance to conventional chemotherapy, and high lethality. Consequently, there is an urgent need to better delineate the molecular pathogenesis of HCC to develop new preventive and therapeutic strategies against this deadly disease. Noticeably, emerging evidence indicates that proteins involved in lipid biosynthesis are important mediators along the development and progression of HCC in humans and rodents. Here, we provide a comprehensive overview of: (a) The pathogenetic relevance of lipogenic proteins involved in liver carcinogenesis, with a special emphasis on the master fatty acid regulator, fatty acid synthase (FASN); (b) The molecular mechanisms responsible for unrestrained activation of FASN and related fatty acid biosynthesis in HCC; (c) The findings in experimental mouse models of liver cancer and their possible clinical implications; (d) The existing potential therapies targeting FASN. A consistent body of data indicates that elevated levels of lipogenic proteins, including FASN, characterize human hepatocarcinogenesis and are predictive of poor prognosis of HCC patients. Pharmacological or genetic blockade of FASN is highly detrimental for the growth of HCC cells in both *in vitro* and *in vivo* models. In conclusion, FASN is involved in the molecular pathogenesis of HCC, where it plays a pivotal role both in tumor onset and progression. Thus, targeted inhibition of FASN and related lipogenesis could be a potentially relevant treatment for human HCC.

## Introduction: Human Hepatocellular Carcinoma

Human hepatocellular carcinoma (HCC) is one of the most frequent and pernicious solid tumors, ranking fifth in incidence and second in lethality worldwide (1–3). Albeit the prevalence of HCC is highest in Eastern Asia and sub-Saharan Africa, where the HBV chronic infection is endemic and the food is contaminated by the mycotoxin aflatoxin B1, its incidence is rapidly rising also in Western Europe and North America (1–3). In the latter areas, however, this escalation in HCC occurrence cannot be entirely explained by the established causal relationship linking chronic hepatitis B or C infection, or ethanol consumption, to hepatocarcinogenesis. Indeed, at least one quarter of HCC cases remains idiopathic (1–3). In the last decade, non-alcoholic fatty liver disease (NAFLD) has emerged for its potential etiopathogenetic role in liver cancer development, especially in industrialized countries. Numerous case-control studies indicate in fact that HCC patients with cryptogenic cirrhosis display clinical and demographic characteristics suggestive of NAFLD, when compared with HCC patients of viral or alcoholic etiology (3–6). In particular, it has been shown that the increased incidence of HCC in the United States over the past few decades has occurred in parallel with the epidemic of NAFLD (3–6). The latter condition is characterized by the excessive accumulation of lipids in the liver and is associated with obesity, insulin resistance, and type 2 diabetes, often evolving into HCC (3–6).

Regardless of the causative agent, most HCC patients are diagnosed with an advanced disease, precluding the employment of potentially curative therapies, including liver transplantation or partial liver resection (1–3). In addition, molecularly based treatments provided negligible benefits in terms of survival in HCC patients, with the multi-kinase inhibitors Sorafenib and Regorafenib being the only drugs able to extend the life expectancy by ~2/3 months (7–9). Consequently, new therapeutic approaches aimed at restraining the growth of advanced HCC are highly needed. For this purpose, the molecular pathogenesis of HCC should be better elucidated to identify critical targets whose inhibition might hamper liver tumor development and/or progression.

## The “Lipogenic Phenotype”

Deregulated lipid biosynthesis (commonly referred to as “*de novo* lipogenesis” or “*de novo* lipid synthesis”) plays an important pathogenetic role in the development of various metabolic diseases, such as diabetes mellitus, obesity, and the metabolic syndrome. In addition, emerging evidences indicate that metabolism reprogramming, including aberrant lipogenesis, is a widespread phenomenon in most cancer types (10–12).

From the historical point of view, the scientific work of the German biochemist and Nobel Prize laureate Otto Warburg, who has been dealing with this issue for several decades since the 1920s, can be considered a pioneer work in this field (13, 14). The starting point was his observation that tumor cells metabolize glucose into lactate under aerobic conditions, while not using the energetically more plausible route of oxidative decarboxylation by the citric acid cycle for energy production. This observation is nowadays well-known as the “Warburg effect” or “Warburg phenomenon” (13, 14). One plausible explanation for this apparently paradoxical event is that glycolysis, although significantly less efficient for energy production than aerobic decarboxylation, can produce adenosine triphosphate (ATP) about 100 times faster than mitochondrial respiration would (14). Consequently, the tumor cell can provide sufficient energy for the accelerated metabolic processes along carcinogenesis. In addition, through the Warburg phenomenon, a reservoir of important metabolic intermediates available for amino acid synthesis and pentose phosphate production—indispensable prerequisites for ensuring adequate protein and DNA synthesis—is generated (14). Furthermore, elevated aerobic glycolysis results in a growth advantage for the most proliferating tumor cells within their microenvironment (14). The immediate consequence of increased glycolysis is the accumulation of the pyruvic acid (pyruvate) metabolite. While most of the pyruvate is converted into lactate and eliminated via the cell membrane, some of the pyruvate is instead converted to acetyl-CoA. In contrast to the normal cell, acetyl-coA represents the primary substrate of the *de novo* lipid synthesis in tumor cells (14).

**Figure 1:**
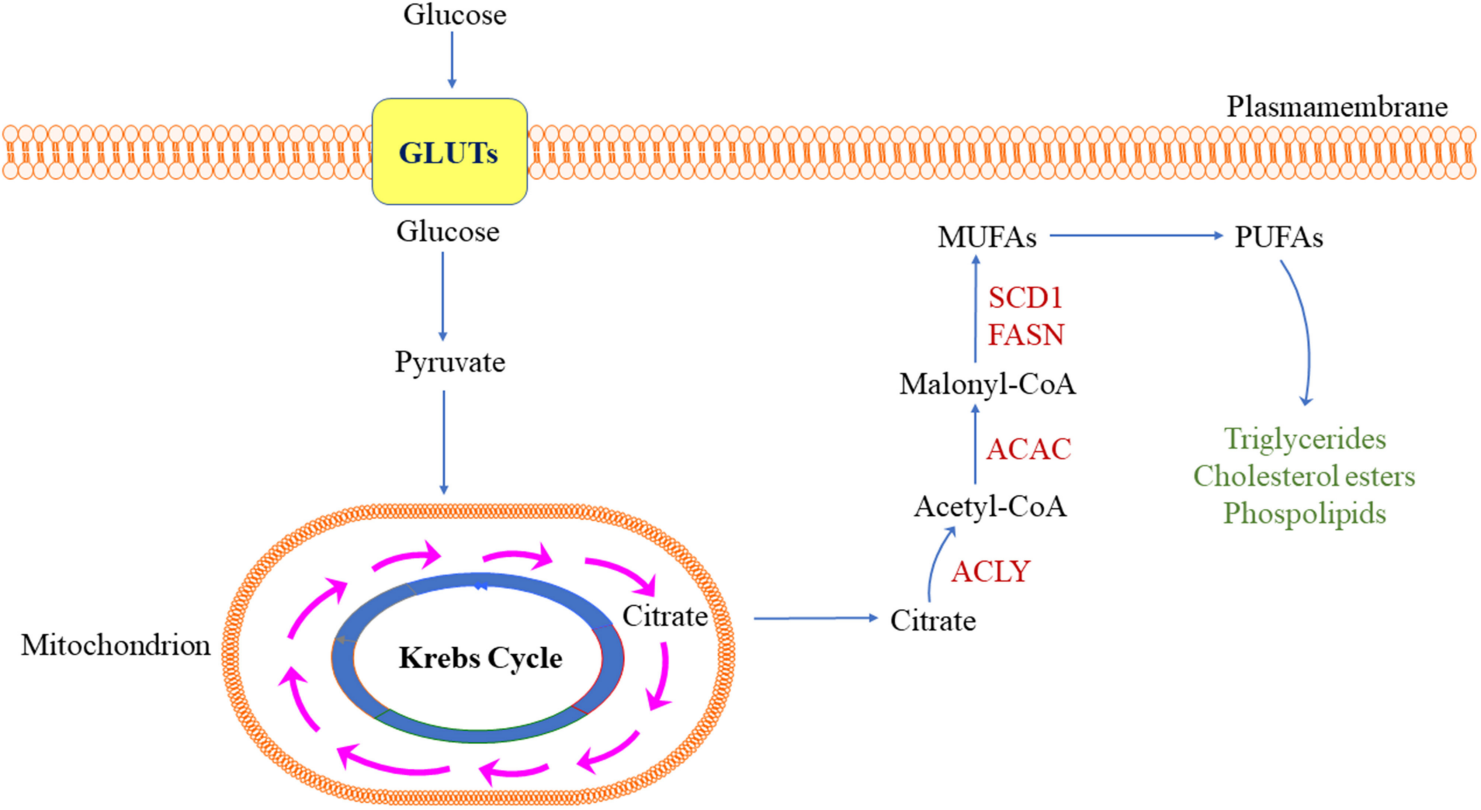
Simplified representation of *de novo* lipogenesis in the tumor cell. ACC, acetyl-CoA carboxylase; ACLY, adenosine triphosphate citrate lyase; FASN, fatty acid synthase; GLUTs, glucose transporters; MUFA, monounsaturated fatty acids; PUFA, polyunsaturated fatty acids; SCD1, stearoyl-CoA desaturase 1. Detailed description of the pathway is reported in the main text.

As normal tissues can cover most of their lipid requirements via dietary lipids coming from the blood circulation, *de novo* lipogenesis does not play a significant role in the metabolism of these cell types; as a result, the expression of lipogenic enzymes is low (10–12). In striking contrast, a universal up-regulation of lipid synthesis occurs in tumor cells (10–12). Importantly, the latter phenomenon is only occasionally associated with a change in cellular morphological properties that are detectable by light microscopy (namely, lipid accumulation in tumor cells that, consequently, appear enlarged, and with a clear cytoplasm) (10–12). Most frequently, indeed, aberrant lipogenesis results in marked alterations of various molecular and metabolic processes, including intracellular signal transduction, and gene expression. At the molecular level, increased lipogenesis is primarily recognizable by the fact that numerous enzymes involved in lipid metabolism (lipogenic enzymes) display strong activity and high expression in tumor cells (10–12). In particular, this refers to the coordinated upregulation of the key enzymes involved in the conversion of glucose into fatty acids, such as ATP citrate lyase (ACLY), acetyl-CoA carboxylase (ACAC), fatty acid synthase (FASN), malate enzyme (ME), and stearoyl-CoA-desaturase 1 (SCD1). Each of these enzymes exhibits a pivotal function in the series of events leading to aberrant lipid biosynthesis. Specifically: (a) ACLY converts citrate from the citrate cycle to acetyl-CoA; (b) ACAC synthesizes malonyl-CoA starting from acetyl-CoA; (c) FASN, starting from malonyl-CoA and consuming acetyl-CoA and NADPH, synthesizes the saturated fatty acid palmitate (palmitic acid) and other saturated long-chain fatty acids; (d) ME catalyzes the production of the reducing NADPH necessary for the synthesis of long-chain fatty acids; and (e) SCD1 converts saturated fatty acids into unsaturated fatty acids, which serve as substrates for the synthesis of triglycerides, cholesterol esters, and phospholipids (10–12, 14, 15). The major steps of *de novo* lipogenesis are summarized in Figure 1.

The requirement of lipids for proliferating tumor cells is high for several reasons. First, lipids represent the building blocks necessary for cell membrane production and, consequently, cell duplication (10–12, 14, 15). Second, the newly synthesized fatty acids are used, if needed, to provide additional energy through the β-oxidation. Third, lipids serve as anchors for selective protein transport to the membrane and as precursors for the synthesis of “lipid second messenger” molecules (10–12, 14, 15).

Based on these data, it is obviously not surprising that most epithelial tumors exhibit an increased *de novo* lipid synthesis and an associated upregulation of the lipogenic enzymes. These include carcinomas of the breast, colorectal, prostate, urinary system, ovary, upper gastrointestinal tract, lung, and oral cavity (10–12). Furthermore, it is well-established that tumor cells display increased ACLY expression and activity. Of note, suppression of ACLY by either small interfering RNA molecules (siRNAs) or the pharmacological inhibitor SB-204990 blunts the proliferation and survival of carcinoma cells both *in vitro* and *in vivo* (16). These intriguing findings are in line with the observation that ACAC, FASN, and SCD1 are up-regulated in numerous malignancies at both the transcriptional and protein level, and their inactivation by treatment with specific siRNAs or small molecular inhibitors significantly restrains tumor cell proliferation and survival (10–12, 14, 15). Altogether, these data suggest that *de novo* lipid synthesis as well as the activation of lipogenic proteins and enzymes are critical for the growth of tumor cells.

## Fatty Acid Synthase In Physiology And Cancer

As reported above, fatty acid synthase (FASN) is the critical enzyme responsible for *de novo* fatty acid synthesis (10–12). Specifically, FASN catalyzes the reaction leading to the generation of palmitate and 16-carbon long fatty acid from acetyl-CoA and malonyl-CoA (10–12). Palmitate is a 16 carbon saturated fatty acid that is a major component of cell membranes and human breast milk, and is incorporated into triglycerides for energy storage. In addition, palmitate is a substrate in the palmitoylation of membrane proteins and acts as a precursor in the synthesis of complex lipids, including cholesterol and glycerophospholipids (10–12).

FASN consists of seven functional domains: acyl carrier protein, malonyl/acetyltransferase, ketoacyl synthase, ketoacyl reductase, dehydrase, enoyl reductase, and thioesterase (17, 18). In humans, FASN is encoded by the *FASN* gene and composed of two identical 272 kDa multifunctional polypeptides, in which the seven domains form a single bond (17). The human *FASN* gene locus is located at chromosome 17 (17q25.3) (10).

FASN is mainly expressed in the cytosol of healthy liver, adipose, brain, cycling endometrium, and lactating mammary gland cells; in these tissues and organs, lipogenesis is a crucial physiological process (10–12).

In cancer, multiple studies have shown that FASN is strongly upregulated in tumors from breast, prostate, colorectal, bladder, ovary, and lung, especially when characterized by clinical aggressiveness, poor prognosis, and resistance to therapy. In contrast, corresponding non-tumor tissues adjacent to the tumor generally express low levels of FASN protein (10–12). However, increased FASN expression has also been detected in some benign and pre-neoplastic lesions of the prostate, breast, lung, stomach, colon, and cutaneous nevi (10–12). Furthermore, investigations conducted in breast, pancreatic, and colorectal tumors showed that cancer patients exhibit elevated levels of FASN in the serum. Once again, FASN levels in patients' serum directly correlate with an adverse outcome (10–12).

Additional evidence linking FASN to cancer comes from experimental models. For instance, *in vitro* ectopic overexpression of *FASN* in breast cancer cells was found to promote lipogenesis along with augmented cell growth and proliferation (19). Also, transgenic overexpression of *Fasn* in mice triggered the development of prostate epithelial neoplasia, albeit it was not sufficient to induce invasive tumors *per se* (20). Further studies with immortalized prostate epithelial cells (iPrEC) suggested that, in addition to the *Fasn* expression, co-expression of androgen receptor was required for invasive adenocarcinoma development (20). Altogether, this body of evidence indicates a unique association between FASN expression and tumor development and/or progression.

## Fatty Acid Synthase In Hepatocellular Carcinoma: Evidence From Human Disease And Experimental Models

The contribution of unrestrained lipogenesis to the development of hepatocellular carcinoma (HCC) and its progression as well as the molecular mechanisms contributing to the aberrant lipid biosynthesis are starting to be understood. Despite the mounting evidence concerning the importance of aberrant lipid biosynthesis in carcinogenesis, the first studies on this phenomenon in human HCC are relative recent. In a small study (21), overexpression of the mRNA of the main lipogenic enzymes (FASN, ACAC, ACLY and SCD1) was described in HCC when compared with non-neoplastic liver counterparts. In addition, the sterol regulatory element-binding protein 1 transcription factor (SREBP1), a major inducer of lipogenesis, has been identified as a negative prognostic factor in liver cancer (22). Also, an *in vitro* study demonstrated that inhibition of FASN significantly affects the growth of human HCC cell lines in a p53-independent manner (23). Based on these intriguing observations, several studies into the pathogenetic relevance of *de novo* lipid synthesis in human HCC have been initiated, especially focusing on the molecular pathways that drive this event.

In a pioneering investigation, we analyzed the levels of the critical lipogenic proteins in a large human HCC collection (24). In particular, the HCC cohort used could be differentiated into two distinct subgroups based on patient survival after partial liver resection: a group of HCC with less aggressive biological behavior or HCCB (defined as survival longer than 3 years) and one with higher aggressive behavior or HCCP (defined as survival time shorter than 3 years) (24). Intriguingly, a simultaneous upregulation of all relevant enzymes of the lipogenic metabolism was observed in HCC when compared with non-tumorous surrounding liver tissues (24). These included the enzymes responsible for fatty acids production (FASN, ACAC, ACLY, ME, and SCD1) as well as the enzymes for cholesterol biosynthesis [SREBP2, 3-hydroxymethylglutaryl-CoA reductase (HMGCR), mevalonate kinase (MVK), and squalene synthetase (SQS)]. Concomitantly, their upstream inducers [carbohydrate-responsive element-binding protein (chREBP), SREBP1, liver X receptor β (LXR-β)] were upregulated. Of note, the highest levels of lipogenic enzymes were detected in HCC with poorer prognosis (HCCP) (24). It is noteworthy to underline that the content of the chemical end products of the respective lipid synthesis (fatty acids, triglycerides, and cholesterol) changed in an analogous manner (24). Thus, these data indicate increased lipogenesis during development and progression of HCC in humans. Subsequent investigations showed that the induction of unrestrained lipogenesis was the result of both transcriptional and post-transcriptional mechanisms (24). Specifically, in addition to the aforementioned transcription factors (chREBP, SREBP1, and LXR-β), we detected a prominent induction of the ubiquitin-specific peptidase 2a (USP2a) (24). This protein is involved in the inhibition of proteasome-induced degradation of FASN, thus inducing stabilization and increased half-life of the latter (25). Similarly, v-akt murine thymoma viral oncogene homologous (AKT) was found to inhibit the ubiquitination of SREBP1 by phosphorylation-dependent mechanisms (24). These findings indicate that presumably a complex program involving several pathways converge to increase lipid biosynthesis in human HCC.

**Figure 2:**
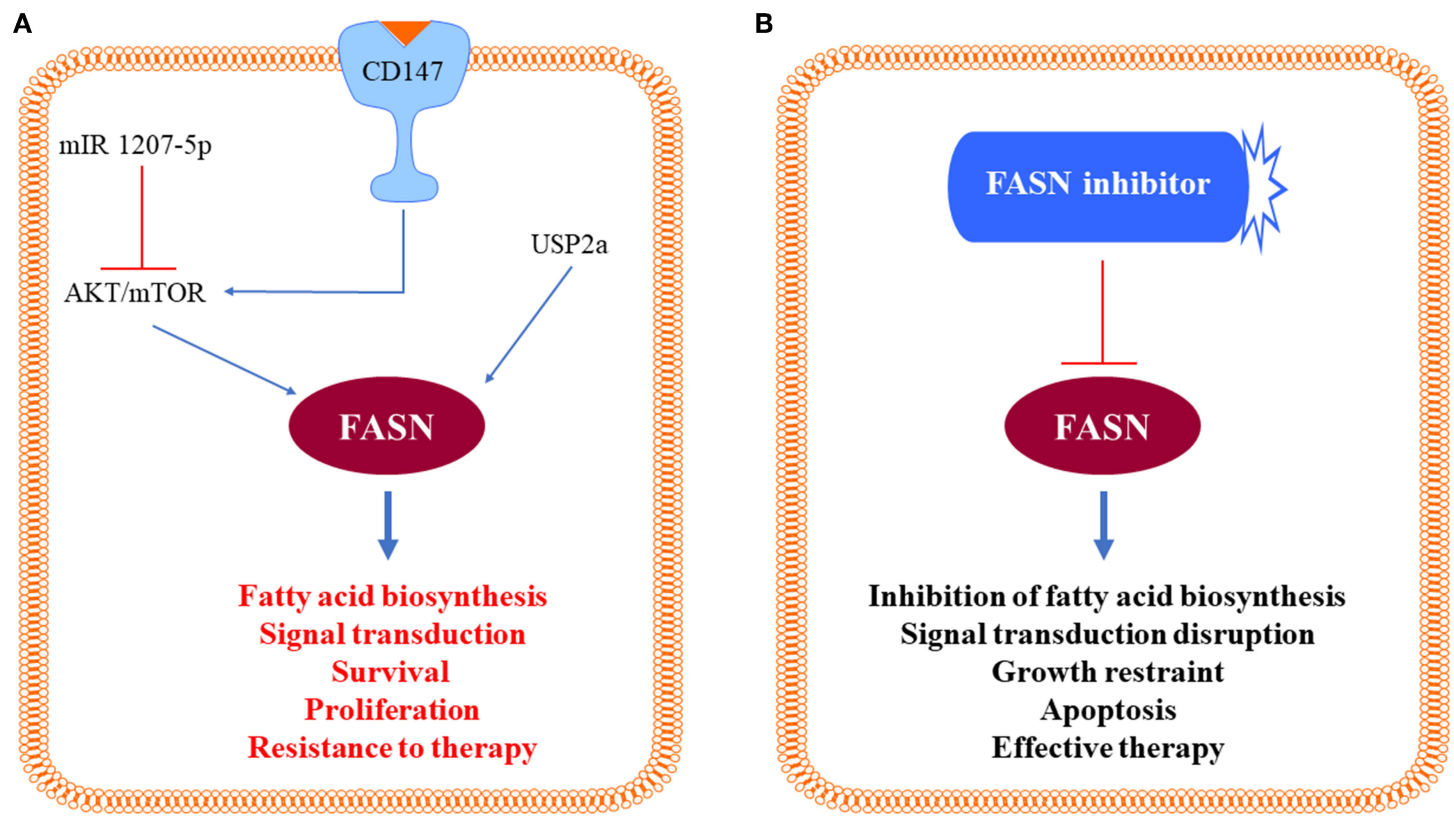
Schematic representation of the identified molecular mechanisms triggering unrestrained fatty acid synthase (FASN) activity in hepatocellular carcinoma cells. **(A)** Positive signals inducing activation of the AKT/mTOR pathway (CD147) and loss of negative stimuli (mIR 1207-5p) toward the same pathway lead to activation of FASN and induction of its multiple, pro-oncogenic biologic effects, which are blunted by FASN inhibitors **(B)**. Further details are reported in the text.

Since it is established that the AKT/mTOR pathway is a prominent inducer of *de novo* lipogenesis in various tissues and organs (26, 27), our group investigated whether this also applies to human HCC. As expected, an increased induction of activated (phosphorylated) AKT, mTOR, and the mTOR effector RPS6, was detected from surrounding liver tissues to HCC, especially HCCP, when compared to normal liver (24). The importance of the AKT/mTOR signaling in lipogenesis was further substantiated in human HCC cell lines, where overexpression of myristoylated/activated AKT led to a rapid increase in cell growth and a reduction in apoptosis. This change in proliferation kinetics was paralleled by a sharp increase in lipid synthesis and up-regulation of lipogenic enzymes in AKT-overexpressing cells (24). Conversely, there was a robust inhibition of cell growth associated with a decrease in lipogenesis and a reduction in the content of lipogenic proteins when AKT was selectively suppressed in HCC cell lines (24). At the molecular level, activation of lipogenesis was dependent on an intact mTOR complex1 (mTORC1)/RPS6 signaling pathway, as the addition of the mTORC1 inhibitor rapamycin or the targeted inactivation of RPS6 by specific siRNA impaired cell growth in the same cell lines (24). The functional importance of the AKT/mTOR pathway in HCC aberrant lipogenesis and FASN induction was substantiated in a recent investigation from Zhao et al. (28). These authors confirmed the relationship between FASN and the AKT/mTOR cascade in HCC cell lines; furthermore, they identified the loss of the microRNA (miR) 1207-5p as a critical mechanism leading to unconstrained AKT/mTOR signaling pathway and FASN activity in human liver cancer (28). Alternatively, activation of the AKT/mTOR/FASN axis might be triggered by upregulation of the basigin/CD147 protooncogene, a molecular event often detected in human hepatocarcinogenesis (29, 30). Taken together, these data indicate that the AKT/mTOR pathway plays a leading role in the activation of lipogenesis in human HCC. The identified molecular mechanisms triggering unrestrained FASN activity and lipogenesis in HCC are summarized in Figure 2.

In light of these important premises, we determined the requirement of FASN and *de novo* lipogenesis in hepatocarcinogenesis *in vivo*, using genetic approaches. To achieve this goal, we employed conditional FASN knockout (KO) mice (31) and various oncogene driven HCC models, such as AKT and AKT/c-Met mice. Previous data from our group showed that hydrodynamic transfection of an activated form of AKT (myristoylated/myr-AKT) triggers upregulation of FASN, aberrant *de novo* lipid synthesis, and HCC development after long latency in mice (24). To determine whether FASN expression is necessary for myr-AKT driven liver tumor development, we hydrodynamically injected myr-AKT and Cre recombinase (AKT/Cre mice) into conditional FASNfl/fl mice (32). Of note, while AKT overexpression in control mice resulted in HCC development within 22–28 weeks post-injection, none of the AKT/Cre mice exhibited pre-neoplastic and neoplastic lesions. Equivalent results were achieved following overexpression of myr-AKT in liver-specific FASN KO mice (AlbCre; FASNfl/fl mice) (32). The anti-neoplastic effect resulting from FASN ablation in AKT/Cre mice was presumably due to the downregulation of rapamycin-insensitive companion of mTOR (Rictor), the critical member of the mammalian target of rapamycin complex 2 (mTORC2) (27), which is responsible for activation of the AKT protooncogene via phosphorylation. The relevance of Rictor in this process was further demonstrated by the finding that genetic depletion of Rictor in hepatocytes prevented myr-AKT driven hepatocarcinogenesis in mice (32). The crucial role of FASN in hepatocarcinogenesis has been confirmed in a second mouse model, where myr-AKT was co-transfected with the protooncogene c-Met (AKT/c-Met mice). In this model, the co-expression of AKT and c-Met was found to dramatically accelerate HCC development in mice when compared to those transfected with AKT or c-Met alone, with all AKT/c-Met mice being required to be euthanized within 8 weeks post-injection due to high tumor burden (33). Thus, AKT, c-Met, and Cre plasmids were transfected into FASNfl/fl mice, allowing the simultaneous expression of AKT and c-Met oncogenes, while deleting FASN in the same subset of mouse hepatocytes (AKT/c-Met/Cre) (33). Once again, genetic inactivation of FASN completely blunted AKT/c-Met-driven hepatocarcinogenesis in AKT/c-Met/Cre mice, implying that although extremely aggressive, AKT/c-Met tumors fully depend on FASN activity to develop (33). Similar results were obtained more recently by Guri et al. (34). These authors generated a mouse model consisting of lack of *Tsc1* and *Pten* tumor suppressor genes, which inhibit the mTORC1 and mTORC2 pathways, specifically in the liver (termed L-dKO mouse). In these mice, liver-specific activation of the mTOR signaling cascade promoted fatty acid synthesis, liver steatosis, and HCC development. Noticeably, either treatment with the FASN inhibitor Orlistat or Fasn knockdown using adenovirus associated virus suppressed hepatocarcinogenesis in L-dKO mice (34).

Altogether, the present data indicate that FASN and related fatty acid biosynthesis play a critical pathogenetic role in hepatocarcinogenesis.

## Inhibition Of Fatty Acid Synthase In Human Hepatocellular Carcinoma: Is It A Feasible Option?

**Figure 3:**
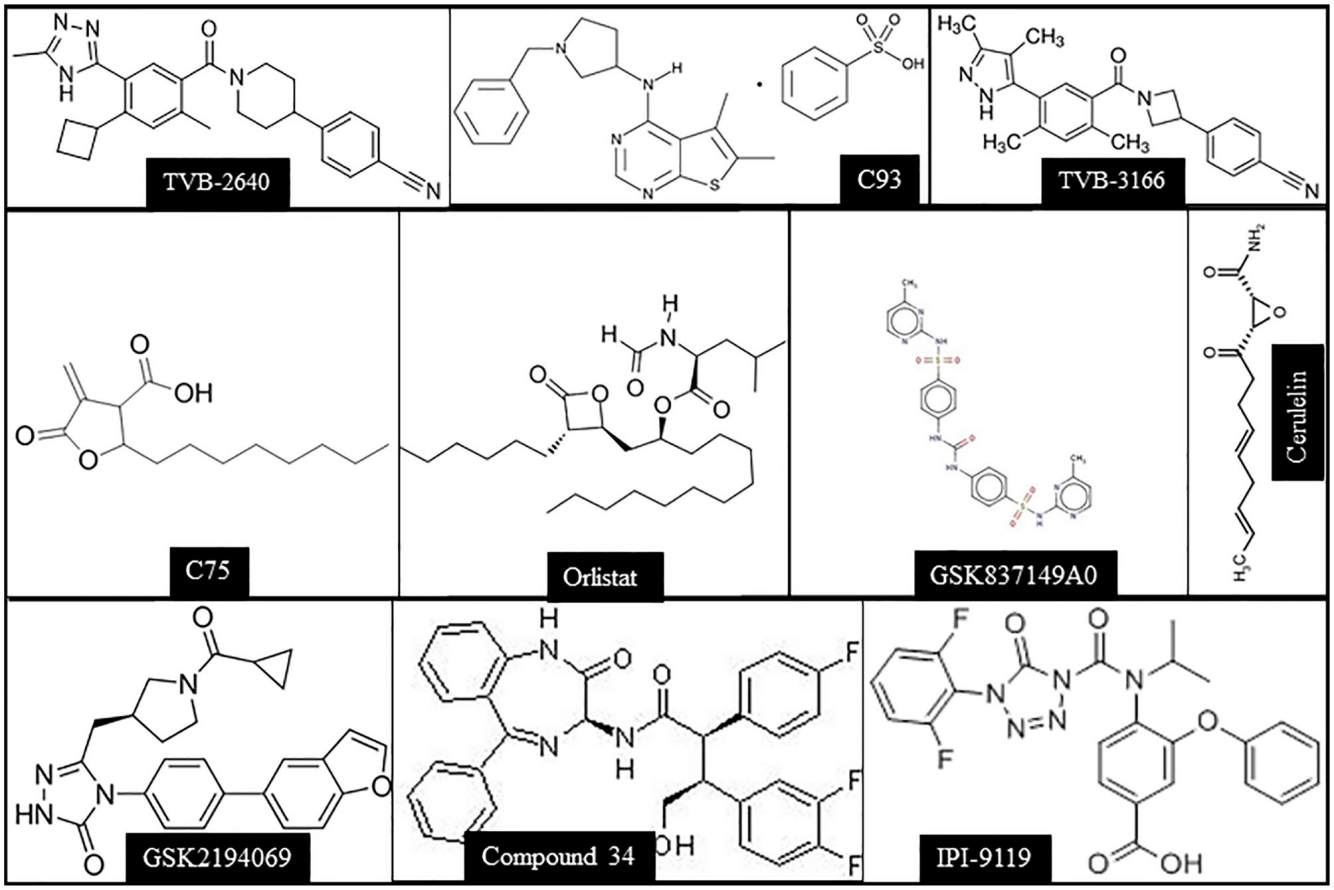
Chemical structures of the main FASN inhibitors tested in preclinical and clinical studies.

Based on the body of evidence presented before, it can be envisaged that FASN inhibition might represent a potentially effective therapeutic strategy against human HCC (35). Several FASN inhibitors have been tested against cancer in preclinical studies, including cerulenin, Orlistat, C75, Fasnall, TVB-2640, and others (Figure 3). However, only TVB-2640 is currently under evaluation, alone or in combination with other medications, in clinical trials against human cancers, not comprising HCC (Table 1).

**Table 1:** Current evidence on the antineoplastic properties of main FASN inhibitors in cancer.

**Name**	**Molecular formula**	**Antineoplastic activity and targeted tumor type**	**Current clinical trials**	**References**
Cerulenin	C_12_H_17_NO_3_	Breast cancer, promyelocytic leukemia and other cells, mouse liver metastases	–	(35, 36)
C75	C_14_H_22_O_4_	Lung cancer cells, radio-sensitization in prostate cancer cells	–	(37–39)
Orlistat	C_29_H_53_O_5_	Prostate, melanoma, breast and other cells, and xenograft tumor models	–	(40–45)
C93	C_13_H_15_NO_5_	Lung, ovarian and trophoblastic neoplasia cells	–	(46, 47)
Fasnall	C_19_H_22_N_4_S·C_6_H_6_O_3_S	Breast cancer (combination therapy)	–	(48)
TVB-3166	C_24_H_24_N_4_O	Lung, ovarian, prostate, and pancreatic xenograft tumor models, combination with taxanes	–	(49, 50)
Compound 34	C_31_H_24_F_3_N_3_O_3_	Ovarian, prostate, prostate, lymphoma, leukemia, myeloma, lung, breast cells		(51)
IPI-9119	C_24_H_19_F_2_N_5_O_5_	Prostate cancer cells	–	(52)
GSK837149A	C_23_H_22_N_8_O_5_S_2_	–	–	(53)
GSK2194069	C_25_H_24_N_4_O_3_	–	–	(35)
JNJ- 54302833	C_30_H_31_N_5_O_2_	–	–	(35)
TVB-2640	C_27_H_29_N_5_O	Numerous solid tumors, several combinations with chemotherapeutic agents under evaluation	NCT03808558 NCT02223247 NCT02980029 NCT03179904 NCT03032484	(55)

*The code of the clinical trials refers to the ClinicalTrials.gov repository*.

Pioneering examples of studies investigating the lipogenic dependency of cancer in recent years include those performed with small molecule FASN inhibitors like cerulenin, an antibiotic isolated from fungal extracts. Cerulenin was found to be active against numerous cancer cell lines and xenograft models; however, the highly reactive nature of the cysteine-reactive epoxide group and off-target activities hampered its clinical application (36). In particular, activation of β-oxidation and excessive energy expenditure, leading to weight-loss or anorexia, represented the major factors limiting the application of cerulenin in humans (35). Similar reasons prevented the clinical use of C-75, a synthetic inhibitor of FASN, which was also demonstrated to possess profound antineoplastic effects in experimental models, and to enhance radiation-induced apoptosis in prostate cancer cells, promoting cell cycle arrest in the G2/M phase (37–39).

Orlistat is an anti-obesity drug, which acts by blocking the absorption of free fatty acids from the gastrointestinal tract through the inhibition of pancreatic and gastric lipase that hydrolyze triglycerides (40, 41). Specifically, Orlistat possesses a highly reactive beta-lactone that covalently captures reactive serine residues in the FASN thioesterase domain (42). Despite its potency in restraining the growth of *in vitro* and *in vivo* cancer models (43, 44), the off-target activities together with the poor water solubility and gastrointestinal absorption have hindered the use of Orlistat as anti-tumor agent in patients (45).

C93 is one of the first inhibitors synthesized, which showed antineoplastic activity initially in lung cancer cell lines, and subsequently in trophoblastic neoplasias (46, 47), but no significant further studies were recently performed. Fasnall, a thiophenopyrimidine-based FASN inhibitor with potent and broad antitumor activity against various breast cancer models, might represent a promising alternative. Fasnall inhibits the FASN capacity to facilitate the production of phospholipids with saturated acyl chains, whereas it promotes the uptake of exogenous unsaturated fatty acids, with consequent alterations in signal transduction messages and promotion of apoptosis. Of note, Fasnall have been shown to act synergistically to prolong the survival of mouse models of breast cancer when associated with the chemotherapeutic agent carboplatin; in this study Fasnall was well tolerated, with no changes in feeding behavior or weight loss being detected in these mice, further suggesting its possible application in the clinical practice (48).

Other high potential FASN inhibitors have been recently developed. Among them, TVB-3166 is a imidazopyridine-based, orally-available, FASN inhibitor, which suppresses *de novo* palmitate synthesis *in vitro* and *in vivo*, and displays antineoplastic activity in several experimental cancer models (49, 50). The mechanism of action of TVB-3166 on aberrant lipogenesis resides on its property to disrupt the architecture of lipid rafts. Alterations in lipid rafts by TVB-3166 promote the mis-localization of membrane-associated oncoproteins, such as Ras, AKT, and members of the canonical Wnt/β-catenin pathway. As a consequence, TVB-3166 administration leads to the abrogation of several signaling cascades and the induction of tumor cell apoptosis (49). Lu et al. have synthesized several FASN inhibitors recently using a structure-based approach guided by X-ray crystallography approach (51). Among them, compound 34 showed a high FASN inhibitory potential and favorable pharmacological features; in addition, it strongly inhibited cell proliferation in several cancer cell lines including A2780 (ovarian), PC3M (prostate), LNCaP (prostate), OCI LY1 (lymphoma), MV4-11 (leukemia/lymphoma/myeloma), H460 (lung), A549 (lung), and MDA-MB-468 (breast), becoming an interesting candidate for future studies (51).

The synthetic drug IPI-9119, which has been recently developed, strongly inhibits FASN by promoting acylation of the catalytic serine, with high selectivity and negligible off-target activity (52). IPI-9119 was shown able to effectively block cell growth and proliferation in several cell lines, including prostatic cancer cells, reducing the proportion of S-phase cells and increased that of G0/G1 cells, and decreasing expression of cyclin A2 (52). GSK837149A was identified as a reversible low inhibitor of the FASN β-ketoacyl reductase domain, but its poor cell permeability prevented the study of its mechanism in cells (53), while other synthetic inhibitors like GSK2194069 and JNJ- 54302833 remain to be tested in pre-clinical models. In addition, several natural plant-derived polyphenols have been shown to inhibit FASN, including epigallocatechin-3-gallate (EGCG) and the flavonoids luteolin, taxifolin, kaempferol, quercetin, and apigenin (54); EGCG in a recent study reduced the growth of adenocarcinoma human lung cancer xenografts without inducing body weight loss (37). Other natural FASN inhibitors may have similar properties, and merit evaluation in future studies.

Currently, the most promising anti-FASN drug is TVB-2640, an oral, small-molecule possessing *in vitro* and *in vivo* antitumor activity associated with an acceptable non-clinical safety profile. Of note, preclinical and early efficacy data from a dose-escalation trial demonstrated a wide activity of TVB-2640 as a single agent in multiple solid tumors, including cases of stable disease (clinicaltrials.gov/ct2/show/NCT02223247). These encouraging results were achieved with relatively low side effects, which could be eliminated with therapy discontinuation (55). Currently four further clinical trials are testing TVB-2640 alone or in combination with other drugs in NSCLC (NCT03808558), colorectal (NCT02980029), breast cancer (NCT03179904), and astrocytomas (NCT03032484).

TVB-2640 combination treatments are based on evidences that FASN inhibitors synergize with multiple chemotherapeutic agents, such as taxanes, vinca alkaloids, 5-fluorouracil, platinum compounds, and anthracyclines. Furthermore, FASN inhibitors have been found to restore the sensitivity to chemotherapeutic drugs, including doxorubicin, and to targeted therapies, such as those including trastuzumab or lapatinib. In addition, FASN suppression might also cooperate in radio-sensitization and with antiangiogenic agents, by triggering strong tumor hypoxia because cancer cells escape antiangiogenic-driven hypoxia by upregulation of FASN-related lipogenesis (38, 56). These evidences strongly suggest that FASN inhibitors will play an important role in future therapeutic attempts against cancer, hopefully also against HCC.

## Conclusion

HCC is a highly aggressive and frequent tumor worldwide, with its incidence rising also in low-frequency areas. Thus, these data, together with the lack of effective therapies against this tumor type, indicate that HCC represents a major health concern globally. Understanding the intricated molecular pathogenesis of this cancer entity is therefore necessary for the identification of specific targets suitable of therapeutic intervention. Recently, among the potential, novel therapeutic targets identified in HCC is FASN and the related *de novo* lipogenesis pathway. Mounting and solid evidence underscores the fact that aberrant fatty biosynthesis contributes to hepatocellular carcinogenesis in experimental models as well as in humans. Albeit several features of FASN and related lipogenesis remain to be explored, it appears clear from the data summarized in the present review article that anti-FASN-based therapies might be helpful for the treatment of HCC treatment. The use of existing drugs against FASN for the treatment of HCC (and other tumors) has been impeded by the low potency and consistent off-target effects of these molecules. However, the most recent FASN inhibitors (e.g. Fasnall, TVB-3166, and TVB-2640) seem to have overcome most of these limitations (56).

Among the critical questions that still need to be addressed for the clinical practice, is how the HCC patients can be selected for anti-FASN treatments. It is clear from HCC TCGA analysis (https://tcga-data.nci.nih.gov/tcga/tcgaHome2.jsp) as well as other genomic studies (57) that human HCC is a highly heterogeneous disease. Not all HCCs express FASN and its related lipogenesis genes at high levels. Consequently, some HCCs might not depend on FASN and *de novo* lipogenesis for growth. This possibility was revealed by *in vivo* mouse studies. Indeed, there was no increase in Fasn expression in mouse HCCs induced by c-Met and gain of function mutant of β-Catenin (c-Met/β-Catenin) via hydrodynamic injection. Consistently, ablation of *Fasn* did not affect HCC growth in mice (35). For this purpose, reliable biomarkers able to uncover the patients who would presumably benefit from this therapeutic strategy should be identified.

Furthermore, as *de novo* lipogenesis is an integrated part of a metabolic network, it is conceivable that disruption of fatty acid synthesis may lead to other biochemical events. These feedback biochemical and metabolic events may contribute to HCC development. For instance, in the diethylnitrosamine (DEN) induced mouse HCC model, inhibition of lipogenesis via deletion of Acac1 and Acac2 genes in the liver led to an increased HCC development (58). Mechanistically, this unexpected finding was due to the marked increased in antioxidants, including increased NADPH and reduced glutathione, which protected hepatocytes from oxidant-mediated cell death. In another example, in murine HCCs induced by overexpression of c-Met and loss of Pten (c-Met/sgPten), loss of Fasn significantly repressed HCC formation. However, over long time, HCC lesions could emerge from Fasn null genetic background. Further molecular and metabolomic analysis revealed that there was an increased cholesterol biosynthesis due to increased Srebp2 activity in the mouse liver tissues. This augmented cholesterogenesis eventually compensated for the loss of *de novo* lipogenesis, ultimately leading to HCC formation (59).

It is also important to acknowledge that two major mechanisms whereby cells acquire fatty acids required for cell growth exist: one involves FASN and its mediated *de novo* lipogenesis, while the other consists of the transport of circulating fatty acids via the “lipolytic” pathway (60). This process requires lipoprotein lipase (LPL), which releases fatty acids from lipoproteins, as well as fatty acid transporter proteins for fatty acids uptake (61). The role of exogenous fatty acids during tumor initiation and progression has been studied marginally to date. However, recent reports suggested the key role of this pathway in tumorigenesis. For instance, it was recently found that fatty acids derived from adipocytes could be transferred to melanoma cells through the fatty acid transporter protein SLC27A1. Blocking fatty acid uptake via the fatty acid transport proteins inhibitor Lipofermata significantly reduced melanoma growth and invasion (62). In HCC cells, it has been shown that LPL mediated fatty acid uptake could at least partly compensate the blockade of *de novo* lipogenesis (63). These studies indicate that presumably both *de novo* fatty acid synthesis and exogenous fatty acid uptake should be inhibited to achieve significant anti-cancer effects.

In addition, future studies are required to determine whether anti-FASN drugs can be used in combination with FDA-approved anti-HCC multi-kinase inhibitors (Sorafenib, Regorafenib, Cabozantinib), immune modulators (checkpoint inhibitors), and/or conventional chemotherapeutic drugs for the treatment of HCC. Studies to address this important point should be conducted. An alternative approach to suppress FASN in HCC (and other tumor types) could be the inhibition of FASN upstream inducers, such as USP2a and CD147. As concerns USP2a, ML364, a small molecule inhibitor of this deubiquitinase has been recently developed. Of note, ML364 administration caused cell cycle arrest in colorectal cancer and lymphoma cell lines, although the specific effect of the drug on FASN levels was not investigated (64). Preliminary results obtained in our laboratory indicate a strong growth restraint as well as downregulation of FASN in HCC cell lines treated with ML364 (Cigliano et al., unpublished observation), suggesting that inhibition of USP2a might be a promising therapy for this deadly disease. Furthermore, targeting CD147 has revealed promising results in the treatment of human HCC patients. Indeed, HCC recurrence rate was found to be significantly decreased, and the survival length of HCC patients subjected to liver transplantation prolonged, following the administration of a monoclonal antibody against CD147, in a randomized controlled trial (65).

## Author Contributions

DC and XC conceived the work, designed the outline of the review, and supervised all aspects of the manuscript. All authors participated in the literature search, scrutiny, and interpretation, as well as in writing and editing all contents of the manuscript.

### Conflict of Interest

The authors declare that the research was conducted in the absence of any commercial or financial relationships that could be construed as a potential conflict of interest.
