# Corrigendum: Pathogenetic, Prognostic, and Therapeutic Role of Fatty Acid Synthase in Human Hepatocellular Carcinoma

**DOI:** 10.3389/fonc.2022.874053

**Published:** 2022-04-12

**Authors:** Li Che, Panagiotis Paliogiannis, Antonio Cigliano, Maria G. Pilo, Xin Chen, Diego F. Calvisi

**Affiliations:** ^1^ Department of Bioengineering and Therapeutic Sciences and Liver Center, University of California, San Francisco, San Francisco, CA, United States; ^2^ Department of Medical, Surgical and Experimental Medicine, University of Sassari, Sassari, Italy; ^3^ Institut für Pathologie, Universität Regensburg, Regensburg, Germany

**Keywords:** hepatocellular carcinoma, *de novo* lipogenesis, FASN, tumor metabolism, precision medicine

In the original article, there was a mistake in **Figure 3** as published. Specifically the structure of Orlistat was incorrect. The corrected **Figure 3** appears below.

**Figure 3:**
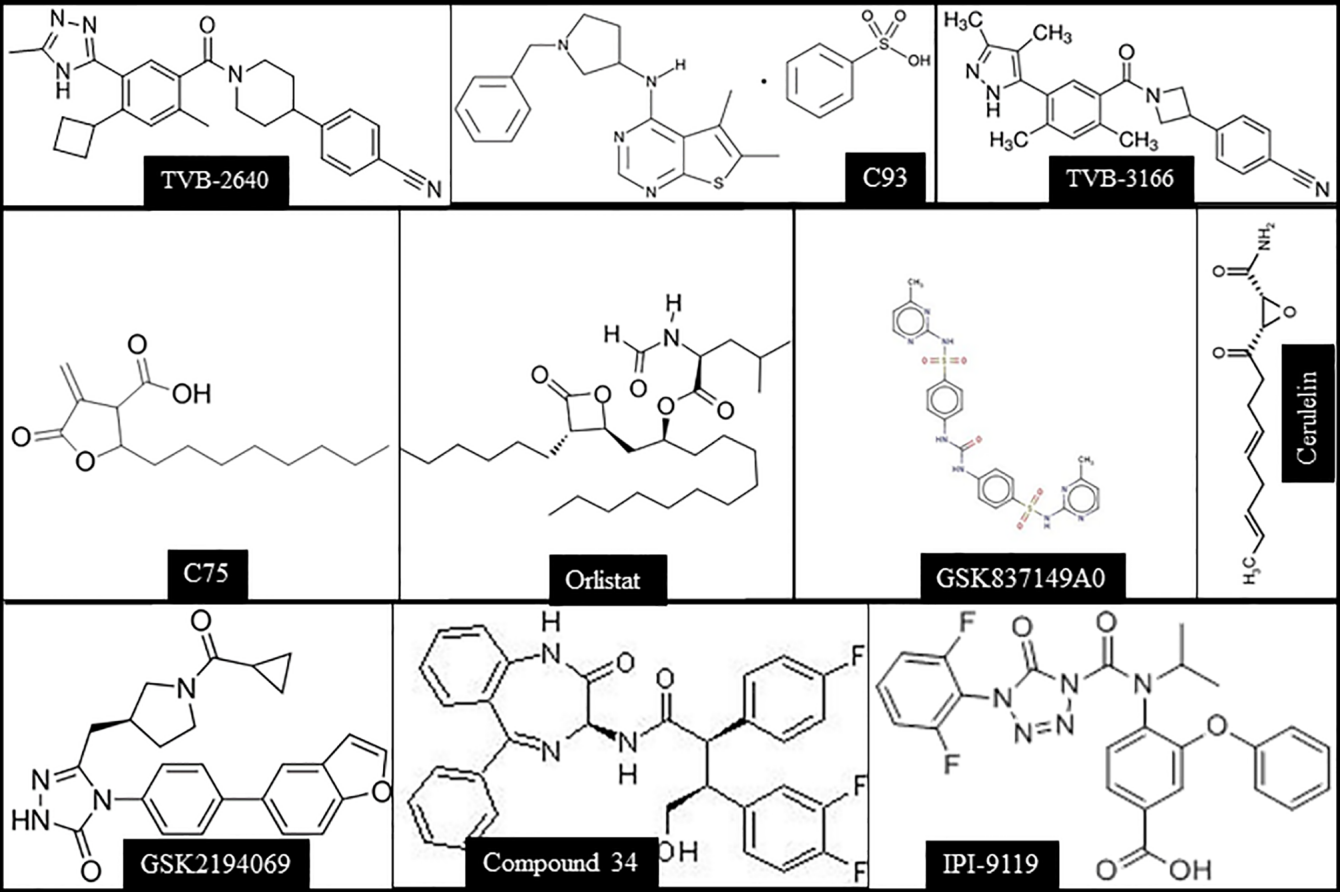
Chemical structures of the main FASN inhibitors tested in preclinical and clinical studies.

The authors apologize for this error and state that this does not change the scientific conclusions of the article in any way. The original article has been updated.

## Publisher’s Note

All claims expressed in this article are solely those of the authors and do not necessarily represent those of their affiliated organizations, or those of the publisher, the editors and the reviewers. Any product that may be evaluated in this article, or claim that may be made by its manufacturer, is not guaranteed or endorsed by the publisher.

